# Gut–Brain Axis in Inflammatory Bowel Disease: Pathogenesis and Therapeutics

**DOI:** 10.26502/aimr.0227

**Published:** 2025-12-10

**Authors:** Samantha Perry, Lekha Pillarisetti, Tamara Gelfman, Devendra K. Agrawal

**Affiliations:** 1 Department of Translational Research, College of Osteopathic Medicine of the Pacific, Western University of Health Sciences, Pomona, California 91766 USA

**Keywords:** Dysbiosis, Fetal microbiota transplantation, Gut-brain axis, Inflammatory Bowel Disease, Microbiome, Neuroimmune signaling, Osteopathic Manipulative Medicine, Osteopathic Manipulative Treatment

## Abstract

Inflammatory Bowel Disease (IBD), encompassing Crohn’s disease and ulcerative colitis, is a chronic inflammatory disorder of the gastrointestinal tract driven by complex interactions between genetic susceptibility, environmental triggers, microbial dysbiosis, and immune dysregulation. The gut microbiome, composed primarily of Firmicutes and Bacteroidetes, plays a crucial role in maintaining intestinal barrier integrity, immune balance, and neuroimmune signaling. Disruption of this microbial ecosystem is characterized by loss of beneficial short chain fatty acid producing bacteria and expansion of pathogenic species which promotes mucosal inflammation, cytokine release, and neuroimmune signaling that can disrupt mental health through the gut-brain axis. Emerging evidence links microbial metabolites, vagal tone, and the hypothalamic-pituitary-adrenal axis in a feedback loop that perpetuates inflammation and alters mood regulation. Current therapeutic approaches include diet modification, osteopathic manipulative treatments, fecal microbiota transplantation and phage therapy. This article focuses on understanding mechanisms linking dysbiosis, immune activation, and neuroinflammation to guide future interventions. A holistic model addressing the gut-brain axis holds the greatest promise for improving outcomes and personalizing care for IBD.

## I. Introduction

Inflammatory Bowel Disease (IBD) represents a chronic, relapsing inflammatory condition of the gastrointestinal tract with significant physical and psychological effects. IBD can have genetic predispositions and environmental triggers that can cause gut microbial imbalances and immune dysregulation [1]. Despite therapeutic advances, disease heterogeneity and unpredictable relapsing complicates management. Studies have shown that both Crohn’s disease and ulcerative colitis, the major forms of IBD, are associated with disruptions to the intestinal epithelium and mucosal immunity that promote sustained inflammation and systemic immune activation [2]. Beyond gut disorders, studies have also shown that SARS-CoV-2–induced ACE2 dysregulation disrupts gut microbiota and the renin–angiotensin system, driving inflammation and neurological dysfunction that contribute to long COVID symptoms [3–7].

Central to IBD pathophysiology is gut microbiome ‘dysbiosis’ which can alter intestinal permeability, immune signaling, and barrier integrity [8]. In healthy individuals, the gut microbiome is predominantly composed of the phyla Firmicutes and Bacteroidetes, which generate short-chain fatty acids (SCFAs) such as butyrate and propionate critical for energy provision and immune regulation. Dysbiosis, characterized by altered microbial diversity and composition, is closely associated with IBD pathogenesis, as evidenced by both experimental models and clinical observations [9]. Loss of anti-inflammatory species and increased pathogenic bacteria heighten mucosal dysfunction and systemic inflammation [10]. In IBD patients, microbial community shifts are evident, marked by a reduction in beneficial Firmicutes, including Faecalibacterium prausnitzii and Roseburia hominis, alongside an increase in potentially pathogenic Proteobacteria and Bacteroidetes. This dysbiotic state correlates with diminished SCFA production and impaired microbial diversity, compromising intestinal barrier function and promoting inflammation. Pathogenic species such as adherent-invasive Escherichia coli, Mycobacterium avium subspecies paratuberculosis, and Listeria monocytogenes have been implicated in disease exacerbation [9]. Whether dysbiosis is the driver of inflammation or result of it remains a central question, but interventional studies with fecal microbiota transplantation and probiotics point to their interrelatedness [8].

Emerging research incorporates the gut–brain axis into IBD frameworks to explain the high rates of mood and cognitive disorders seen in patients [11]. Neuroimmune signaling, vagal pathways, and stress-driven hypothalamic pituitary adrenal (HPA) activation connect gut inflammation to altered brain function and behavior [1]. Circulating cytokines and microbial metabolites can affect glial and neural pathways, linking intestinal pathology to depression, anxiety, and cognitive deficits [2]. Thus, understanding the molecular and inflammatory relationship between the gut and brain provides a foundation for IBD management that targets microbiota composition, immune modulation, and psychological health.

## II. Genetic Susceptibility, Environmental Factors, and Immune System Interactions in IBD

The pathogenesis of IBD results from the convergence of host genetic susceptibility, environmental triggers, immune dysregulation, and microbial perturbation. Genome-wide association studies have identified more than 240 IBD-related loci, many of which encode proteins involved in innate immune sensing, epithelial barrier function, and autophagy [12]. Genes such as NOD2, ATG16L1, CDC42, and ORMDL3 contribute to microbial recognition and mucosal homeostasis; their dysregulation promotes exaggerated immune responses to commensal bacteria [13,14]. Recent work from Agrawal and colleagues highlights CDC42 and ORMDL3 regulatory patterns as potential therapeutic targets for controlling mucosal and metabolic inflammation in IBD [13,15,16].

Environmental influences– diet, antibiotic exposure, infections, early-life microbial colonization– further shape disease risk by altering microbial composition and immune tolerance [17]. Diets low in fiber and high in animal fat reduce microbial diversity and the production of anti-inflammatory SCFAs such as butyrate, propionate, and acetate. Conversely, plant-based diets enrich SCFA-producing genera and support epithelial barrier integrity [18]. Bacaloni and Agrawal emphasized how nutritional patterns and epigenetic modification jointly modulate the microbiota and immune signaling, illustrating how diet serves as a bridge between genetic predisposition and immune activation [19].

Immunologically, IBD involves chronic activation of Th1/Th17 responses, inflammasome signaling, and impaired regulatory T-cell activity. The NLRP3 inflammasome, in particular, connects dysbiosis and metabolic inflammation to intestinal and systemic pathologies [20]. Malicevic and colleagues demonstrated that NLRP3 activation contributes to both IBD and diabetes via gut microbial alterations, suggesting a shared inflammatory circuitry [15,16]. Such systemic inflammation has neuropsychiatric consequences: cytokines like IL-6, TNF-ɑ, and IL-1β cross the blood-brain barrier, modulate neurotransmitter metabolism, and induce microglial activation, contributing to mood disorders [21].

## III. Mechanistic Pathways Linking Gut Inflammation and Mental Health

### 1. Barrier dysfunction and immune activation

In both IBD and IBS, epithelial barrier breakdown permits translocation of bacterial antigens and metabolites, stimulating mucosal and systemic inflammation [22]. Microbial components activate pattern recognition receptors (TLR4, NOD2) and inflammasomes, generating pro-inflammatory cytokines that act on the CNS. These cytokines alter neuronal plasticity and neurotransmission, paralleling mechanisms observed in major depressive disorder [23].

### 2. Vagal and neuroendocrine signaling

The vagus nerve provides a rapid conduit between gut and brain. Vagal afferents detect microbial metabolites and inflammatory mediators, influencing limbic circuits regulating mood. Reduced vagal tone–common in IBD and chronic stress– is linked to greater anxiety and depressive symptoms [24]. In parallel, activation of the HPA axis elevates cortisol, disrupts barrier function, and modifies microbial composition, creating a feedback loop between stress and inflammation [25].

### 3. Metabolic and neurotransmitter pathways

Microbial metabolites such as SCFAa, secondary bile acids, and tryptophan derivatives modulate CNS function. Dysbiosis in IBD/IBS decreases SCFA production, compromising energy supply to colonocytes and anti-inflammatory signaling [26]. Altered bile acid metabolism impacts farnesoid X receptor (FXR) and TGR5 pathways, which influence mood and cognition.

A critical biochemical node is the tryptophan-kynurenine pathway. Inflammatory activation diverts tryptophan metabolism away from serotonin synthesis towards kynurenine and quinolinic acid, both of which can be neurotoxic [27]. This shift contributes to depressive phenotypes in inflammatory states. Experimental manipulation of gut microbiota in rodents modulates tryptophan metabolism and behavioral outcomes, underscoring causality [28].

### 4. Microglial and astrocytic activation

Peripheral inflammation signals to the brain through cytokines and microglia. Chronic exposure leads to sustained microglial activation and reduced neurogenesis in the hippocampus– pathophysiological hallmarks of depression [29]. Animal models of colitis show that microbial or cytokine modulation can reverse these CNS changes [30].

## III. Dysbiosis and Inflammation in IBD: Cause or Consequence

The mystery of whether dysbiosis causes intestinal inflammation or results from it remains unsolved. Longitudinal and gnobiotic studies offer clues. In germ-free mice colonized with microbiota from IBD patients, recipients develop low-grade inflammation and anxiety-like behaviors, implying a causal role for microbial factors [31]. Conversely, induction of colitis alters microbial communities, supporting a bidirectional model [32].

Human cohort studies show that microbial diversity reduction often precedes clinical flares in Crohn’s disease and ulcerative colitis, predicting relapse risk [33]. Similarly, in intestinal bowel syndrome (IBS), psychosocial stress and altered gut-brain signaling precede dysbiosis, suggesting a top-down component [34]. Therefore, the relationship is circular: inflammation modifies the microbiota, which in turn perpetuates inflammation and psychological distress.

Emerging data from other inflammatory conditions reinforce this model. Pathak and Agrawal’s article of long-COVID described gut microbial perturbations correlating with neuropsychiatric symptoms, mirroring gut-brain-immune dynamics of IBD [3–7]. Such parallels support a systems-level view in which dysbiosis and chronic inflammation drive multi-organ consequences, including mental health changes.

## IV. Targeted Therapeutic Strategies

### Probiotics and Prebiotics

A probiotic is a live microorganism while a prebiotic is a non-digestible food ingredient that selectively stimulates the growth of beneficial microorganisms in the gut. A heavily interconnected network that frames a major component of the gut-brain axis is the gut microbiota, making it a reasonable target for therapy. In COVID-19 patients, there was a statistically significant improvement in remission rate in probiotic treated patients compared to controls [35]. Prebiotics such as lactulose, lactosucrose, oligofructose, and inulin have been found to induce the growth of certain host microflora resulting in enriched enteric function [36]. A combination of synergistically acting prebiotics and probiotics, known as synbiotics are supposed to selectively stimulate growth and/or activation of the metabolism of intestinal microbiota positively affecting the host. Adults IBS patients had a significant reduction in the severity of IBS symptoms with multi-strain synergistic therapy [37]. There is rising evidence indicating the modulation of prebiotics and probiotics on the human gut microbiota and its alterations towards a healthier composition for patients [38].

### Dietary Interventions

The diet has the strongest and most direct influence over the microbiota of the gut profile [33]. It is a risk factor for pathophysiology and a therapy for active disease. A common diet recommended to patients with IBD is the healthy Mediterranean diet rich in fresh fruits and vegetables, monounsaturated fats, complex carbohydrates and lean protein [39].

### FMT (Fecal Microbiota Transplantation)

Fecal Microbiota Transplantation is the reestablishment of a healthy gut microbiome, promoting the growth of certain species and preventing the colonization of others by infusing a fecal inoculation from a healthy donor into a patient. This is most effective for patients who have recurrent Clostridium difficile infections. There are current clinical trials being conducted to evaluate FMT as a new therapy in IBD. However, there has been a lack of consistency so far, indicating more research needs to be completed to identify the optimal microbiota composition for long term efficacy in IBD patients [40].

### Phage Therapy

Phage therapy is the modulation of phageome and bacteriome of a person suffering from a disease of bacterial origin [41]. It has grown in popularity since it can be used as an alternative treatment when traditional antibiotics are ineffective in patients with IBD. The addition of bacteriophages to the human microbiome can target the elimination of harmful pathogens without disturbing the beneficial microbial communities. The low toxicity minimizes risk of severe immune reactions and adverse effects, and the self-replication allows low doses to be effective [42].

### Osteopathic Manipulative Treatment Considerations

While there has not been much research conducted on the effects of osteopathic manipulative treatment (OMT) on IBD specifically, multiple studies have suggested that OMT can improve inflammation [43]. OMT demonstrated a reduction in TNF-ɑ in patients with chronic low back pain [44]. Additionally, OMT has shown to promote balance of the autonomic nervous system by attenuating sympathetic activity and increasing parasympathetic activity [43]. These ideas can be applied to reducing the increase in cytokines occurring with IBD patients. Various soft non-manipulative treatments on Crohn’s disease including cranial, myofascial and visceral techniques improved overall and physical quality of life [45].

## V. Future Directions and Conclusion

IBD arises from a complex interplay of genetic susceptibility, immune dysregulation, environmental influences, and gut microbial imbalance. Dysbiosis and chronic inflammation extend beyond the gut, affecting neural pathways that regulate mood and cognition. Cytokine signaling, impaired mucosal function, and altered microbiome metabolites disrupt serotonin synthesis and contribute to CNS symptoms frequently observed in IBD. This bidirectional relationship between inflammation and the CNS reinforces disease persistence and impacts quality of life. Similarly gut, brain, and immune interactions seen in conditions like long COVID highlight shared systemic mechanisms. Dietary interventions, particularly the Mediterranean diet, can positively shape gut microbiota and serve as therapy for IBD. Emerging treatments such as fetal microbiota transplantation and phage therapy aim to restore healthy gut balance and target harmful bacteria, though more research is needed for consistent efficacy. OMT may also benefit IBD patients by reducing inflammation, balancing autonomic activity, and improving quality of life through gentle, non-invasive techniques. Effective management requires an integrative approach that targets both the gut and CNS through microbiome and neuroimmune therapies. Future research should clarify the causal links between dysbiosis, immune activation, and CNS outcomes to guide more precise, personalized treatment options. Understanding the gut brain axis will be key to developing personalized and holistic treatment strategies for IBD.

## Figures and Tables

**Figure 1:**
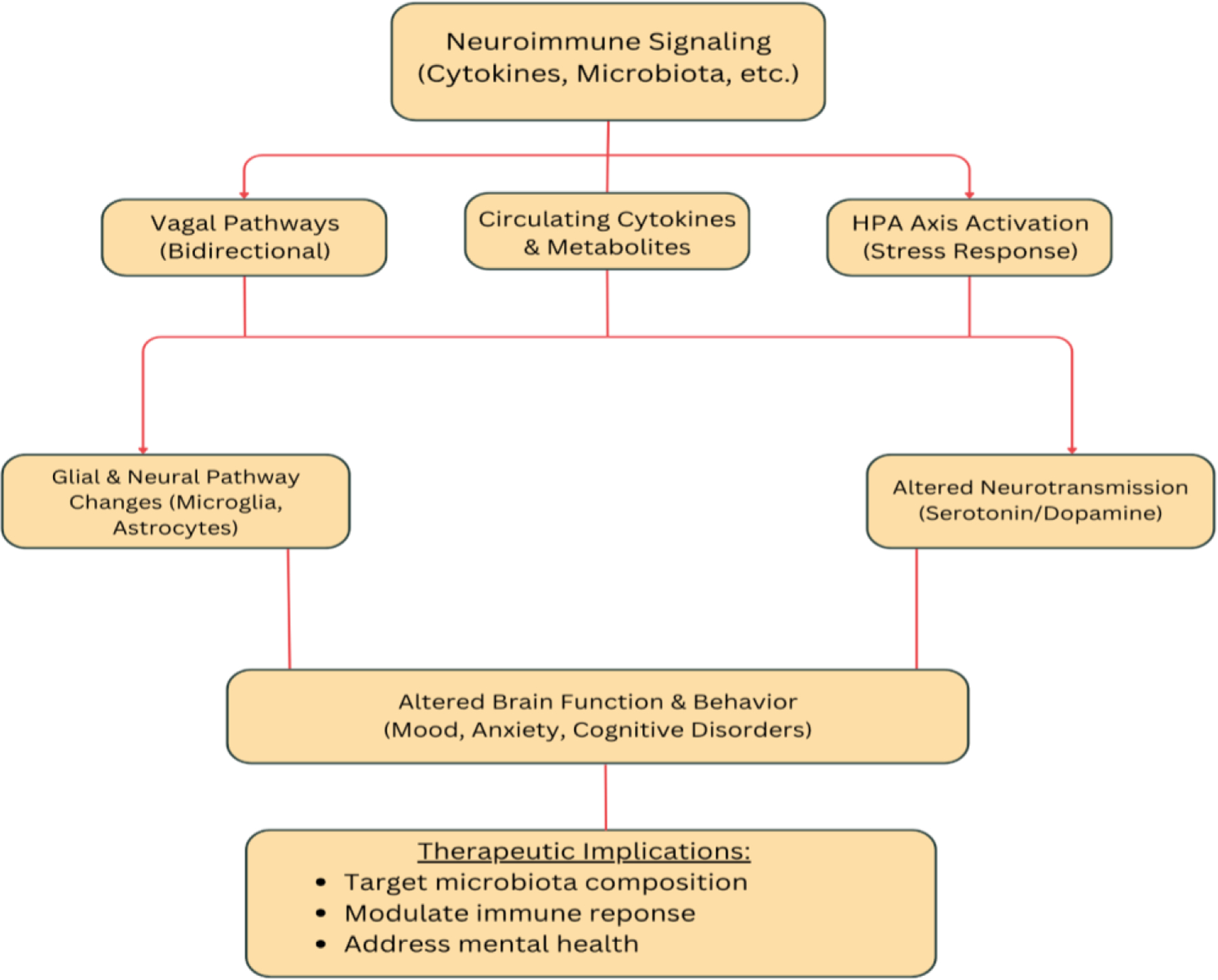
This flowchart depicts the neuroimmune signaling involved in the gut-brain axis and downstream effects that implicate the mental health and microbiome symptoms.

## VI. Key points

- Inflammatory Bowel Disease (IBD) is a chronic inflammatory disorder driven by intestinal barrier disruption, immune dysregulation, and systemic inflammation.
- Dysbiosis in IBD involves loss of beneficial bacteria and expansion of pathogens, reducing SCFA production and impairing gut integrity.
- Over 240 genetic loci, including NOD2 and ATG16L1, link host susceptibility to impaired microbial sensing and immune regulation.
- Environmental factors like diet, antibiotics, and infections modulate the microbiome and influence IBD onset and severity.
- Chronic activation of Th1/Th17 cells and NLRP3 inflammasome drives mucosal and systemic inflammation.
- Gut inflammation communicates with the brain through cytokine, vagal, and HPA-axis pathways, contributing to depression and anxiety in IBD.
- Altered microbial metabolites disrupt serotonin and kynurenine pathways, promoting neuroinflammation and mood changes.
- Treatments such as probiotics, prebiotics, fetal microbiota transplantation, phage therapy, and Mediterranean diets aim to restore microbial balance and reduce inflammation.
- There is a gap in our knowledge concerning whether there is a causal effect of dysbiosis on the chronic inflammation, but current evidence suggests a bidirectional relationship.
- Future research must clarify causal links between dysbiosis, immune activation, and CNS effects to enable personalized treatments.

## References

[1] Carloni S, Rescigno M. The gut-brain vascular axis in neuroinflammation. Semin Immunol 69 (2023): 101802. 37422929 10.1016/j.smim.2023.101802

[2] Agirman G, Yu KB, Hsiao EY. Signaling inflammation across the gut-brain axis. Science. 374 (2021): 1087–1092. 34822299 10.1126/science.abi6087

[3] Pathak A, Agrawal DK. Role of Gut Microbiota in Long COVID: Impact on Immune Function and Organ System Health. Arch Microbiol Immunol 9 (2025): 38–53. 40051430 PMC11883900

[4] Lopes LA, Agrawal DK. Thromboembolism in the Complications of Long COVID-19. Cardiol Cardiovasc Med 7 (2023): 123–128. 37389402 10.26502/fccm.92920317 PMC10310316

[5] Zadeh FH, Wilson DR, Agrawal DK. Long COVID: Complications, Underlying Mechanisms, and Treatment Strategies. Arch Microbiol Immunol 7 (2023): 36–61. 37388279 PMC10310313

[6] Wais T, Hasan M, Rai V, Agrawal DK. Gut-brain communication in COVID-19: molecular mechanisms, mediators, biomarkers, and therapeutics. Expert Rev Clin Immunol 18 (2022): 947–960. 35868344 10.1080/1744666X.2022.2105697 PMC9388545

[7] Thankam FG, Agrawal DK. Molecular chronicles of cytokine burst in patients with coronavirus disease 2019 (COVID-19) with cardiovascular diseases. J Thorac Cardiovasc Surg 161 (2021): e217–e226. 10.1016/j.jtcvs.2020.05.083 PMC7834736 32631657

[8] Hillestad EMR, van der Meeren A, Nagaraja BH, Gut bless you: The microbiota-gut-brain axis in irritable bowel syndrome. World Journal of Gastroenterology 28 (2022): 412–431. 35125827 10.3748/wjg.v28.i4.412 PMC8790555

[9] Santana PT, Rosas SLB, Ribeiro BE, Dysbiosis in Inflammatory Bowel Disease: Pathogenic Role and Potential Therapeutic Targets. Int J Mol Sci 23 (2022). 10.3390/ijms23073464 PMC8998182 35408838

[10] Santos DA, Galiè S. The Microbiota–Gut–Brain Axis in Metabolic Syndrome and Sleep Disorders: A Systematic Review. Nutrients 16 (2024): 390. 38337675 10.3390/nu16030390 PMC10857497

[11] Socała K, Doboszewska U, Szopa A, The role of microbiota-gut-brain axis in neuropsychiatric and neurological disorders. Pharmacological Research 172 (2021): 105840. 34450312 10.1016/j.phrs.2021.105840

[12] Jostins L, Ripke S, Weersma RK, Host-microbe interactions have shaped the genetic architecture of inflammatory bowel disease. Nature 491 (2012): 119–124. 23128233 10.1038/nature11582 PMC3491803

[13] Stojanovic M, Agrawal DK. CDC42 Regulatory Patterns Related to Inflammatory Bowel Disease and Hyperglycemia. J Bioinform Syst Biol 8 (2025): 17–28. 40183002 PMC11967731

[14] Chu H, Khosravi A, Kusumawardhani IP, Gene-microbiota interactions contribute to the pathogenesis of inflammatory bowel disease. Science 352 (2016): 1116–1120. 27230380 10.1126/science.aad9948 PMC4996125

[15] Malicevic U, Rai V, Skrbic R, Agrawal DK. Modulation of Orosomucoid-like Protein 3 Activity in the Management of Inflammatory Bowel Disease. J Biotechnol Biomed 7 (2024): 433–444. 39619146 10.26502/jbb.2642-91280167 PMC11606571

[16] Malicevic U, Rai V, Skrbic R, Agrawal DK. NLRP3 Inflammasome and Gut Dysbiosis Linking Diabetes Mellitus and Inflammatory Bowel Disease. Arch Intern Med Res 7 (2024): 200–218. 39328924 10.26502/aimr.0178 PMC11426418

[17] Zuo T, Kamm MA, Colombel JF, Urbanization and the gut microbiota in health and inflammatory bowel disease. Nat Rev Gastroenterol Hepatol 15 (2018): 440–452. 29670252 10.1038/s41575-018-0003-z

[18] Makki K, Deehan EC, Walter J, Bäckhed F. The Impact of Dietary Fiber on Gut Microbiota in Host Health and Disease. Cell Host Microbe 23 (2018): 705–715. 29902436 10.1016/j.chom.2018.05.012

[19] Bacaloni S, Agrawal DK. Nutrition, Gut Microbiota, and Epigenetics in the Modulation of Immune Response and Metabolic Health. Cardiol Cardiovasc Med 9 (2025): 111–124. 40443829 PMC12121961

[20] Zhen Y, Zhang H. NLRP3 Inflammasome and Inflammatory Bowel Disease. Front Immunol 10 (2019): 276. 30873162 10.3389/fimmu.2019.00276 PMC6403142

[21] Miller AH, Raison CL. The role of inflammation in depression: from evolutionary imperative to modern treatment target. Nat Rev Immunol 16 (2016): 22–34. 26711676 10.1038/nri.2015.5 PMC5542678

[22] Turner JR. Intestinal mucosal barrier function in health and disease. Nat Rev Immunol 9 (2009): 799–809. 19855405 10.1038/nri2653

[23] Dantzer R, O’Connor JC, Freund GG, From inflammation to sickness and depression: when the immune system subjugates the brain. Nat Rev Neurosci 9 (2008): 46–56. 18073775 10.1038/nrn2297 PMC2919277

[24] Bonaz B, Bazin T, Pellissier S. The Vagus Nerve at the Interface of the Microbiota-Gut-Brain Axis. Front Neurosci 12 (2018): 49. 29467611 10.3389/fnins.2018.00049 PMC5808284

[25] Moloney RD, Desbonnet L, Clarke G, The microbiome: stress, health and disease. Mamm Genome 25(2014): 49–74. 24281320 10.1007/s00335-013-9488-5

[26] Parada Venegas D, De la Fuente MK, Landskron G, Short Chain Fatty Acids (SCFAs)-Mediated Gut Epithelial and Immune Regulation and Its Relevance for Inflammatory Bowel Diseases. Front Immunol 10 (2019): 277. 30915065 10.3389/fimmu.2019.00277 PMC6421268

[27] Kennedy PJ, Cryan JF, Dinan TG, Clarke G. Kynurenine pathway metabolism and the microbiota-gut-brain axis. Neuropharmacology 112 (2017): 399–412. 27392632 10.1016/j.neuropharm.2016.07.002

[28] Zheng P, Zeng B, Zhou C, Gut microbiome remodeling induces depressive-like behaviors through a pathway mediated by the host’s metabolism. Mol Psychiatry 21 (2016): 786–796. 27067014 10.1038/mp.2016.44

[29] Yirmiya R, Goshen I. Immune modulation of learning, memory, neural plasticity and neurogenesis. Brain Behav Immun 25 (2011): 181–213. 20970492 10.1016/j.bbi.2010.10.015

[30] Wang L, Li M, Dong Y, Magnoflorine alleviates colitis-induced anxiety-like behaviors by regulating gut microbiota and microglia-mediated neuroinflammation. Microbiome 13 (2025): 172. 40713743 10.1186/s40168-025-02158-y PMC12297679

[31] Kelly JR, Borre Y, C OB, Transferring the blues: Depression-associated gut microbiota induces neurobehavioural changes in the rat. J Psychiatr Res 82 (2016): 109–118. 27491067 10.1016/j.jpsychires.2016.07.019

[32] Frank DN, Amand AL St, Feldman RA, Molecular-phylogenetic characterization of microbial community imbalances in human inflammatory bowel diseases. Proc Natl Acad Sci U S A 104 (2007): 13780–13785. 17699621 10.1073/pnas.0706625104 PMC1959459

[33] Halfvarson J, Brislawn CJ, Lamendella R, Dynamics of the human gut microbiome in inflammatory bowel disease. Nat Microbiol 2 (2017): 17004. 28191884 10.1038/nmicrobiol.2017.4 PMC5319707

[34] Moloney RD, Johnson AC, O’Mahony SM, Stress and the Microbiota-Gut-Brain Axis in Visceral Pain: Relevance to Irritable Bowel Syndrome. CNS Neurosci Ther 22 (2016): 102–117. 26662472 10.1111/cns.12490 PMC6492884

[35] Wais T, Hasan M, Rai V, Agrawal DK. Gut-brain communication in COVID-19: molecular mechanisms, mediators, biomarkers, and therapeutics. Expert Rev Clin Immunol. 2022; 18 (2022): 947–960. 35868344 10.1080/1744666X.2022.2105697 PMC9388545

[36] Roy S, Dhaneshwar S. Role of prebiotics, probiotics, and synbiotics in management of inflammatory bowel disease: Current perspectives. World J Gastroenterol 29 (2023): 2078–2100. 37122604 10.3748/wjg.v29.i14.2078 PMC10130969

[37] Skrzydło-Radomańska B, Prozorow-Król B, Cichoż-Lach H, The Effectiveness of Synbiotic Preparation Containing Lactobacillus and Bifidobacterium Probiotic Strains and Short Chain Fructooligosaccharides in Patients with Diarrhea Predominant Irritable Bowel Syndrome-A Randomized Double-Blind, Placebo-Controlled Study. Nutrients 12 (2020). 10.3390/nu12071999 PMC7400954 32635661

[38] Simon E, Călinoiu LF, Mitrea L, Vodnar DC. Probiotics, Prebiotics, and Synbiotics: Implications and Beneficial Effects against Irritable Bowel Syndrome. Nutrients 13 (2021). 10.3390/nu13062112 PMC8233736 34203002

[39] Hashash JG, Elkins J, Lewis JD, AGA Clinical Practice Update on Diet and Nutritional Therapies in Patients With Inflammatory Bowel Disease: Expert Review. Gastroenterology 166 (2024): 521–532. 38276922 10.1053/j.gastro.2023.11.303

[40] Elhag DA, Kumar M, Saadaoui M, Inflammatory Bowel Disease Treatments and Predictive Biomarkers of Therapeutic Response. Int J Mol Sci 23 (2022). 10.3390/ijms23136966 PMC9266456 35805965

[41] Maronek M, Link R, Ambro L, Gardlik R. Phages and Their Role in Gastrointestinal Disease: Focus on Inflammatory Bowel Disease Cells 9 (2020). 10.3390/cells9041013 PMC7226564 32325706

[42] Li Y, Li XM, Duan HY, Advances and optimization strategies in bacteriophage therapy for treating inflammatory bowel disease. Front Immunol 15 (2024): 1398652. 38779682 10.3389/fimmu.2024.1398652 PMC11109441

[43] Gillan R, Bachtel G, Webber K, Osteopathic manipulative treatment for chronic inflammatory diseases. J Evid Based Med 17 (2024):172–186. 38488211 10.1111/jebm.12590

[44] Licciardone JC, Kearns CM, Hodge LM, Minotti DE. Osteopathic manual treatment in patients with diabetes mellitus and comorbid chronic low back pain: subgroup results from the OSTEOPATHIC Trial. J Am Osteopath Assoc. 113 (2013): 468–78. 23739758

[45] Espí-López GV, Inglés M, Soliva-Cazabán I, Serra-Añó P. Effect of the soft-tissue techniques in the quality of life in patients with Crohn’s disease: A randomized controlled trial. Medicine (Baltimore) 97 (2018): e13811. 30572544 10.1097/MD.0000000000013811 PMC6320155

